# Cardiac Cell Therapy for Heart Repair: Should the Cells Be Left Out?

**DOI:** 10.3390/cells10030641

**Published:** 2021-03-13

**Authors:** Dashuai Zhu, Ke Cheng

**Affiliations:** 1 Department of Molecular Biomedical Sciences and Comparative Medicine Institute, North Carolina State University, Raleigh, NC 27607, USA; dzhu4@ncsu.edu; 2 Joint Department of Biomedical Engineering, University of North Carolina at Chapel Hill & North Carolina State University, Raleigh, NC 27607, USA

**Keywords:** stem cell, myocardial infarction, cardiovascular diseases, acellular therapy, exosomes, paracrine effects, biomedical engineering, biomaterials

## Abstract

Cardiovascular disease (CVD) is still the leading cause of death worldwide. Coronary artery occlusion, or myocardial infarction (MI) causes massive loss of cardiomyocytes. The ischemia area is eventually replaced by a fibrotic scar. From the mechanical dysfunctions of the scar in electronic transduction, contraction and compliance, pathological cardiac dilation and heart failure develops. Once end-stage heart failure occurs, the only option is to perform heart transplantation. The sequential changes are termed cardiac remodeling, and are due to the lack of endogenous regenerative actions in the adult human heart. Regenerative medicine and biomedical engineering strategies have been pursued to repair the damaged heart and to restore normal cardiac function. Such strategies include both cellular and acellular products, in combination with biomaterials. In addition, substantial progress has been made to elucidate the molecular and cellular mechanisms underlying heart repair and regeneration. In this review, we summarize and discuss current therapeutic approaches for cardiac repair and provide a perspective on novel strategies that holding potential opportunities for future research and clinical translation.

## 1. Introduction

Cardiovascular disease (CVD) is still the leading cause of death across the world, accounting for nine million deaths per year in the United States alone [1]. According to the 2017 National Health Interview Survey, the age-adjusted prevalence of all types of heart disease was 10.6% [2], of which coronary artery disease and myocardial infarction (MI) were the most common types. Occlusion of the coronary artery causes massive loss of cardiomyocytes (CM) in the heart. As the endogenous regeneration ability of the adult human heart is quite limited, injury is eventually replaced by a fibrotic scar that lacks the essential electronic transduction, contraction, and compliance of viable cardiac muscles. The sequential process of fibrotic scar formation is termed cardiac remodeling, which leads to pathological cardiac dilation and fatal heart failure. Once end-stage heart failure develops, heart transplantation is the only therapeutic option.

Clinical treatment of ischemic heart diseases focuses on cardiac protection and prevention of further occlusion. For example, timely thrombolysis, artery reopening, and bypass surgery have been established to improve blood supply and to salvage injured myocardium. Consequently, thrombolytic agents, antiplatelet drugs, and vasodilators, as well as angioplasty have been developed. Moreover, beta-blockers, angiotensin-converting enzyme (ACE) inhibitors, angiotensin receptor–neprilysin inhibitors, and mineralocorticoid receptor antagonists are prescribed in pharmacological approaches to suppress cardiac remodeling [3,4,5,6]. However, these treatments cannot overcome the substantial loss of functional heart muscles, and therefore limited physical activity and routine medication are required in the daily lives of the patients. Even so, patients can still develop heart failure with the currently available therapeutic options.

In order to repair and regenerate functional tissues or organs, researchers have focused on stem cells and regenerative medicine strategies. Specifically, biomaterials and tissue engineering have been gaining attention. Cardiac biomedical engineering, which aims at the reestablishment of the structure and functional features of the native heart, was originally developed from the transplantation of cells and organs [7]. Multiple types of stem cells, such as skeletal blast cells (SKBCs), bone marrow-derived mononuclear cells (BM-MNCs), endothelial progenitor cells (EPCs), hematopoietic stem cells (HSCs), mesenchymal stem cells (MSCs), embryonic stem cells (ESCs), induced pluripotent stem cells (iPSCs), cardiac progenitor cells (CPCs), and cardiosphere-derived cells (CDCs) have been studied. Additionally, numerous biomaterials and tissue engineering scaffolds have been fabricated by researchers worldwide to increase the retention and survival rates of transplanted cells. Lastly, entering the new era of cardiac stem cell therapy, stem cells derivatives such as exosomes, secretome, microRNAs, and synthetic stem cell-mimicking materials have been tested [8,9].

Inspired by regenerative medicine and biomedical engineering strategies, novel approaches to repair cardiac damage and restore cardiac function, including cellular and acellular therapies, have shown substantial progress in understanding the molecular and cellular mechanisms underlying heart regeneration. In this review, we discuss current therapeutic approaches for cardiac repair and regeneration, and we summarize the detailed mechanisms of these therapeutics with the hope of providing prospection on potential opportunities for future research and clinical translation focused on heart regeneration.

## 2. Cellular Therapy for Heart Regeneration and Repair

The first step in repairing an injured heart, is the replacement of scar tissue with viable heart muscle tissue. However, it has been well established that the heart lacks innate regenerative ability. Endogenous renewal, mitosis, and division of cardiomyocytes occur only during embryonic, fetal development, and neonatal stages [10]. In rodents, there is a brief postnatal window of seven days for cardiac regeneration [11,12]. Genetic fate mapping indicates that the majority of regenerated cardiomyocytes originate from preexisting cardiomyocytes [12]. In pigs, the regenerative window shrinks after two postnatal days [13]. It is still unclear whether this regenerative capacity exists in humans. In addition, there is a lack of evidence for cardiac stem cell identification in mammalians. Nonetheless, because of stem cells’ differentiation potential to form other lineage cells, stem cell therapy has been broadly explored for cardiac repair and cardiac regeneration. Stem cells ranging from skeletal blast cells (SKBCs), embryonic stem cells (ESCs), adult stem cells (such as MSCs, EPCs, and HSCs), cardiosphere-derived cells (CDCs), and induced stem cells (iPSCs) have been used for treatment of heart injury.

### 2.1. Induced Pluripotent Stem Cells (iPSCs)

In 2006, Yamanaka and colleagues reported the reprogramming of mouse and human fibroblast to an ESC-like pluripotent state by forced expression of *Oct3*/*Oct4*, *Sox2*, *Klf4*, and *Myc* genes. This reprogrammed cell was named induced pluripotent stem cells (iPSCs) [14]. ESCs and iPSCs are both pluripotent stem cells that can differentiate into all kinds of tissues in the body. However, the application of ESCs has been met with ethical concerns. In contrast, iPSCs, which can be induced from autogenous fibroblast of the patient, have gained great interest in regenerative applications.

The discovery of iPSCs offered a regenerative approach for damaged heart repair. In culture, iPSCs can be induced to generate large numbers of functional cardiomyocytes. By temporal modulation of the expression of regulators in the canonical Wnt signaling pathway, iPSCs can be induced to yield a high purity (80–98%) of cardiomyocytes in 14 days [15,16]. iPSC-derived cardiomyocytes (iPSC-CMs) were transplanted for treatment of MI in a rodent model [17,18,19,20,21]. After transplantation, engrafted iPSC-CMs improved ventricular function and reduced pathological remodeling [17,20,21]. Moreover, pretreatment with Y-27632, an inhibitor of Rho-associated protein kinase (ROCK) [18], or genetic overexpression of the cell cycle activator cyclin D2 (*CCND2*) [19], enhanced the regenerative potency of iPSC-CMs by stimulating proliferation, contraction, and suppressing apoptotic death [18,19]. In a clinically relevant non-human primate model of ischemia/reperfusion injury, direct intramyocardial (IM) injection of iPSC-CMs improved cardiac contractile function at 4 and 12 weeks after transplantation. The grafted cells exhibited electrical coupling with host cardiomyocytes as assessed by use of the fluorescent calcium indicator G-CaMP7.09 [22]. In addition, the incidence of ventricular tachycardia was detected in iPSC-CMs injected monkeys [22], and in another study, using human ESC-derived cardiomyocytes, post-transplant arrhythmias were also recorded [23]. Despite the replenishment of cardiomyocytes in infarcted myocardium with iPSC-CMs implantation, the engraftment rate is still low because of immune rejection. The concerns of ventricular arrhythmias have also been highlighted.

To address this problem of low engraftment and arrhythmia, Ye et al. [24] fabricated a cardiac muscle patch by combining iPSC-derived cardiovascular lineage cells (cardiomyocytes, endothelial cells, and smooth muscle cells). Four weeks after epicardial implantation, iPSC-derived cardiomyocytes integrated into host myocardium and generated organized sarcomere structure. The engrafted endothelial cells and smooth muscle cells contributed to host vasculature. Most importantly, by using the epicardial cardiac patch, they achieved cardiac regeneration without inducing ventricular arrhythmias [24]. Furthermore, they engineered functional human cardiac muscle patches (hCMPs) with clinically relevant dimensions (4 cm × 2 cm × 1.25 mm), which were implanted to treat pig MI [25]. Four weeks after transplantation, measurements of cardiac function, infarct size, and wall stress were significantly improved with no increase in the occurrence of arrhythmogenic complications [25]. Patching up the heart has been proposed to be an efficient way to restore functional myocardium by using stem cell-based therapy [26,27,28]. However, the reported benefits of transplanted cells have been attributed to, paracrine effects in large part, instead of to the direct replacement of infarct scar by viable cardiomyocytes [29].

### 2.2. Cardiac Progenitor Cells (CPCs)

Even though the adult heart lacks regenerative abilities, cellular turnover occurs in the heart [30], and the existence of a cardiac progenitor cell population has been reported [31,32,33]. Different than terminally differentiated cardiomyocytes, CPCs are highly proliferative and have differentiation potential [31,34]. CPCs represent another kind of stem cell that is used for cardiac repair. Various approaches have been developed to increase CPC populations [35,36,37] due to their extremely low levels in the heart [37].

CPCs are identified by detection of specific markers, such as c-kit [38], Sca-1 [39], Mesp1 [40], and Flk-1 [31]; c-kit+ CPCs have been broadly employed for MI treatment [38,41,42]. Although c-kit+ CPCs cannot differentiate into cardiomyocytes [43,44,45,46], transplantation of c-kit+ CPCs has been reproducibly shown to be beneficial in both preclinical and clinical studies for heart disease treatment [42,47,48,49,50]. These reports imply that c-kit+ CPCs are working via paracrine effects. Stem cell antigen-1 (Sca-1) positive cells are also defined as CPCs [51,52,53,54]. When implanted into ischemia-damaged mice myocardium, Sca-1+ CPCs differentiated into endothelial-like cells and promoted cardiac reparation [51,53]. By using a genetic lineage tracing method, Ronald J. Vagnozzi et al. [55] reported that Sca-1+ cells reveal endothelial but not myogenic contribution to the murine heart. In response to pathological stress, Sca-1+ CPCs expand and preferentially contribute to vasculature. In other studies, the absence of cardiomyocytes, differentiated from Sca-1+ cells, has also been reported [56,57]. Nevertheless, other than the differentiation of vascular cells, Sca-1+ CPCs can also exert cardioprotective roles by secreting bioactive factors [58].

Flk1+ cells and Mesp1+ cells represent another two types of CPCs identified in heart [33,59,60]. Both of them direct cardiovascular genesis during embryonic development. Flk1 is expressed in multipotent mesodermal progenitor cells, which is specified to form myocardial and endothelial lineages during development [61]. Through genetic fate mapping, researchers have demonstrated that Flk1+ cell populations contribute to progenitors that have differentiation potentials to cardiomyocyte, endothelial, and vascular smooth muscle cell [31], but there was no proof for preclinical and clinical regenerative efficacy. Mesp1 is a transcription factor that drives cardiovascular progenitor cell specification. Transient expression of Mesp1 increases the number of cardiac progenitor cells in heart [40,62,63]. Mesp1 can directly bind to the promoter region of the transcription factors known to direct cardiomyocyte differentiation. These transcription factors include Hand2, Gata4, Nkx2.5, Myocardin, FoxH1, and FoxC1 [40]. After in vitro induction from ESCs, Mesp1+ cells were injected into hearts of MI mice. The results showed that Mesp1+ CPCs emerged de novo into terminally differentiated cardiac myocytes, smooth muscle cells, and vascular endothelial cells, accompanied by significant functional improvement in mice [60].

In addition to the above intrinsic types, CPCs can be induced to generate from iPSCs and ESCs. These cells are named induced cardiac progenitor cells (iCPCs) [35,36]. In contrast to the direct implantation of iPSCs and ESCs, which pose the risks of tumorgenesis or teratomas [64], iCPCs are safer for application because of their restricted differentiation potential. Moreover, iCPCs are expandable (can be passaged more than 18 times), which limits cost and opens additional avenues for clinical translational use of patient-specific cell sources [65].

### 2.3. Cardiosphere-Derived Cells (CDCs)

Given the notion that tissue-derived cells hold potential for regeneration and repair [66], patient cardiac tissues can be acquired and cultured, and then yield spherical multicellular clusters called cardiospheres [67,68]. Culturing cardiospheres produces cardiosphere-derived cells (CDCs), which are used for regenerative therapy for MI [69,70,71,72,73]. In culture, CDCs showed myogenic differentiation and angiogenic potential [74]. After intramyocardial injection, CDCs preserved ventricular ejection function and attenuated cardiac remodeling [71,75]. In 2012, the first clinical trial of CADUCEUS (CArdiosphere-Derived aUtologous stem CElls to reverse ventricUlar dySfunction) [76,77] explored the safety and efficacy of intracoronary infusion of autologous CDCs. Preliminary indications included decreased scar size, increased viable myocardium, and improved regional function of infarcted myocardium [77]. These studies showed attractive benefits of using CDCs for MI treatment. However, CDCs cannot differentiate toward cardiomyocytes. The underlying therapeutic effects of CDCs have been attributed to paracrine factors [74,78].

### 2.4. Mesenchymal Stem Cells (MSCs)

MSCs are a population of stromal cells that are isolated from various kinds of tissues, such as adipose, placenta, and bone marrow. In culture, MSCs can be induced to differentiate exclusively into the adipocytic, chondrocytic, or osteocytic cell lineages [79]. These intrinsic differentiation properties are usually used for the identification or characterization of MSCs [80]. Moreover, MSCs can be identified by detecting the expression of surface marker CD73, CD90, and CD105, and the lack of expression of hematopoietic lineage markers CD45, CD34, CD11b, CD79, and HLA-DR [81].

Because of the wide distribution of MSCs throughout the body and the multipotent differentiation actions, MSCs are the most commonly used cell type in regenerative medicine for the treatment of various types of diseases, including myocardial infarction [82]. In vitro studies have demonstrated the differentiation potential of MSCs into cardiac lineage cells when media is supplemented with additional factors such as 5-azacytidine, bone morphogenetic protein-2 (BMP-2), angiotensin-II, dimethyl sulfoxide, and fibroblast growth factor-4 [83,84]. In addition, co-culture with MSCs protects cardiomyocytes from oxidative stress [85]. In preclinical studies [86,87,88,89,90], in vivo delivery of MSCs contributed to vascular regeneration and inflammation resolution in the infarcted myocardium, as well as improvement of left ventricular ejection function. Mounting data acquired from basic research has driven the implementation of clinical trials [91,92]. In a randomized, double-blind, placebo-controlled, dose-escalation, parallel-assigned clinical trial, the safety and efficacy of intravenous infusion of bone marrow MSCs for the treatment of acute MI was shown [86]. Furthermore, intravenous injection of human umbilical cord MSCs have been shown to be safe and effective for improving cardiac perfusion and function [93,94].

The abovementioned data recapitulate the therapeutic applications of MSCs for treatment of MI. However, there are also concerns regarding the therapeutic efficacy. The diverse sources of MSCs have resulted in different efficacy yields in similar studies [95]. Meanwhile, MSCs cannot form cardiomyocytes after implantation. As with other stem cell types, the potential mechanisms underlying MSCs therapy are broadly accepted as paracrine effects [95].

### 2.5. Other Types of Cells

In addition to the cell types emphasized above, there are also other cell types, such as skeletal blast cells (SKBCs), bone marrow mononuclear cells (BMMNCs) [47], bone marrow-derived progenitor cells [96], endothelial progenitor cells (EPCs), and hematopoietic stem cells (HSCs) [97] that have been used for treatment of heart injury. SKBCs share the same contractile and sarcomere features as cardiomyocytes. but do not integrate electrically with intrinsic cardiomyocytes after implantation, and the clinical trials had been terminated due to arrhythmic events [98]. Clinical studies using BMMNCs for MI treatment have also show either no or small improvements in cardiac performance [99]. EPCs cannot differentiate into cardiomyocytes in vivo, but they do play roles in promoting angiogenesis and contribute to vasculature. EPCs can also provide cardiomyocytes with survival signals via paracrine effects [100]. HSCs do not have the ability to generate functional cardiomyocytes, and the reported efficacy has been attributed to paracrine effects.

Even though cellular therapy has been expected with cardiac muscle regeneration, except for pluripotent stem cells (ESCs and iPSCs) and iCPCs, none of the other cells has regenerated a functional heart in practice. With stem cell engraftment, there are also concerns of immune rejection and cell loss. Faced with the failure of heart regeneration, paracrine mechanisms have been proposed to explain therapeutic effects.

## 3. Paracrine Effects

Despite the expectation that cellular treatment would replenish functional cardiomyocytes in the infarct, the engraftment rate is low due to immune rejection. On-going research has highlighted paracrine effects by which stem cells secret bioactive components to modulate angiogenesis, proliferation/mitosis, inflammation, apoptosis, and fibrosis, as well as the other aspects involved in the pathophysiological processes of cardiac repair [101]. Other than the direct differentiation, paracrine effects have become the detailed mechanisms underlying the therapy of multiple types of stem cells as mentioned above.

### 3.1. Promotion of Angiogenesis

Angiogenesis, also known as neovascularization, is the process by which new vasculature is generated from pre-existing blood vessels in response to pathophysiological stimuli [102]. In the setting of MI, ischemic insult destroys intrinsic blood vessels, and subsequent formation of new capillaries has been shown to promote cardiac repair [103]. In recent advances of stem cell-based therapy, promotion of angiogenesis is a key goal. Notably, MSCs are propagates to promote angiogenesis and restoration of ischemic tissues [104]. MSCs can participate in angiogenesis via direct differentiation, cellular contact interaction, and secreting pro-angiogenic factors, of which paracrine actions are considered to be the principal mechanism [105]. By analyzing the cytokine profile in secretome of umbilical cord MSCs, a series of pro-angiogenic factors, including VEGF, IGF-1, and IL-8 have been identified [106]. Moreover, MSCs have been found to stimulate angiogenic activities in endothelial cells by the activation of VEGF-A signaling pathway via secreting endothelin-1, IL-8, platelet-derived growth factor-AA (PDGF-AA), and IGF-2 [107].

In addition to the secretion of proangiogenic factors, exosome transfer from MSCs to target cells can induce angiogenesis-related cellular activities. Exosomes are extracellular vesicles with a size ranging from 40 to 160 nm in diameter [108]. They carry nucleic acids, proteins, lipids, and metabolites, and are mediators of intercellular communication. MSC exosomes promote angiogenesis by delivering microRNAs and protein factors. Various microRNAs, including microRNA-21 (miR-21), miR-126, miR-155, and let-7a, have been characterized in MSC exosomes that mediate capillary-formation behavior in endothelial cells [109,110]. MiR-21 induces tube-forming capacity of primary bovine retinal endothelial cells by highlighting the expression of VEGF [111] and miR-126 benefits vascular integrity and regulate angiogenic signaling in endothelial cells [112]. Expression oflet-7a promotes endothelial cell proliferation and motility [113]. Taken together, the abovementioned research shows the pro-angiogenic activities of MSCs mediated by paracrine effects.

### 3.2. Suppression of Inflammation

Numerous studies have confirmed the immunosuppressive effects of MSCs in promoting inflammatory resolution [114,115]. PGE2 is a lipid signaling molecule that prevents the maturation of dendritic cells and inhibits the proliferation of cytotoxic T cells [116]. Moreover, PGE2 participates in the repair of tissue injury by stimulating angiogenesis [117]. It has been reported that MSCs maintain their immunoprivilege by secreting high levels of PGE2, which suppresses cytotoxic T-cell proliferation and promotes the production of regulatory T-cell by inducing CCL12 and CCL5 secretion [118]. TSG-6 has been reported to play a positive role in treatment of MI with MSCs [119]. TSG-6 is highly expressed in MSCs in response to injury, which subsequently leads to increased extracellular secretion of TSG-6 protein. By activating alpha-1, a serine protease inhibitor, TSG-6 reduces cardiac inflammation [120].

Macrophages modulate the inflammatory microenvironment during injury. In the first phase of MI, infiltration of macrophage (M1 macrophage) elicits a release of proinflammatory cytokines that exacerbate the ischemic injury [121]. Following the clearance of necrotic debris, the macrophage changes its phenotype into the pro-reparative, and becomes an M2 macrophage [121,122]. Accelerating the transition of macrophage from M1 to M2 has been reported to be cardiac protective [122]. Several cytokines that are reported to promote the transition of macrophage phenotypes can be secreted by MSCs, such as IL-10 and TGF-β [123]. IL-10 is an anti-inflammatory cytokine that plays a critical role in the control of immune responses [124]. Most importantly, IL-10 has been reported to trigger changes in macrophage phenotype by promoting anti-inflammatory gene (*Arg1*, *Mrc1* and *Tgfb1*) expression, which facilitates heart wound healing and improves cardiac performance [125].

### 3.3. Promotion of Survival and Proliferation

Numerous studies have repeatedly demonstrated the decrease of apoptotic cell counts in injury after treatment with MSCs or MSCs-conditioned medium (MSC-CM) [126,127]. The PI3K/Akt signaling pathway is crucial for regulating cell cycling, mitosis, and proliferation while suppressing apoptosis. In a mouse model of renal injury, treatment with MSC-CM increased the phosphorylation of Akt [127], which led to the dissociation of Bcl-2-associated cell death promoter (BAD) proteins and eventually suppressed apoptosis. Transplantation of hypoxia-pretreated MSCs enhanced morphological and functional improvements in infarcted hearts by increasing expression of Bcl-2 and its receptor Bcl-xL. Upregulation of Bcl-2 and Bcl-xL have been shown to prevent cell death and apoptosis under hypoxic conditions [128]. Conditioned medium from hypoxia-pretreated Akt-modified MSCs (Akt-MSCs) markedly inhibited hypoxia-induced apoptosis of adult rat cardiomyocytes. After in vivo infusion, Akt-MSCs significantly limited infarct size and improved cardiac performance as compared with controls [129]. Moreover, conditioned medium, from heat shock protein (HSP)-20, overexpressing MSCs, protected adult rat cardiomyocytes against oxidative stress via enhanced activation of Akt and increased secretion of growth factors VEGF, FGF-2, and IGF-1 [130]. To summarize, the abovementioned studies show the protective roles of MSCs on preventing apoptotic cell death through modulation of the PI3K/Akt and MAPK signaling pathways via paracrine effects [131]. Nevertheless, more studies are still required to elucidate the detailed mechanisms.

Coinciding with the activation of anti-apoptotic pathways, cellular proliferation machinery can be activated [127]. Co-culture of MSCs with cisplatin-treated renal epithelial cells has been shown to significantly increase the expression of growth factors such as FGF, EPGF, VEGF, and HGF [132]. These growth factors promoted proliferation of intrinsic endothelial cells and fibroblasts. Moreover, MSCs recruit endogenous stem cell homing to an injury site by aid of SDF-1/CXCL4 axis. On the one hand, endogenous MSCs sense the SDF-1 gradient, proliferate, and migrate towards damage [133,134]. On the other hand, MSCs secret SDF-1 to recruit endogenous cardiac stem cells [135]. These actions further accelerate the shift of the microenvironment from damaged to reparative prone.

### 3.4. Other Aspects of Paracrine Effects

Beside the modulation on angiogenesis, inflammation, and cell proliferation, there are also other aspects that can be regulated by paracrine effects, such as differentiation of endogenous stem cells, extracellular matrix homeostasis, antifibrosis, and chemoattraction. Because of the complexity of the paracrine components, the activities involved in paracrine effects are yet to be elucidated.

## 4. Acellular Therapy

Regenerative medicine aims to restore damaged or malfunctioning tissue through cell-based therapies. However, the challenges of cell loss and poor engraftment caused by immune rejection have made acellular therapy an alternative. The beneficial roles of paracrine factors have made cell-free therapy an important aspect of regenerative medicine. In cell-free therapy, the bioactive executors of paracrine effects, including growth factors, exosomes, and microRNAs, are employed to protect the heart from disease progression [8]. Leaving out viable stem cells, cell-free therapy provides a clinically feasible, easy-to-store alternative manner by reducing the challenges previously listed that arise with live cells. Moreover, biomimetic design of cell-free therapeutics through a bioengineering method adds more advanced properties to acellular therapy.

Since stem cells exert therapeutic effects by paracrine bioactive components, the secreted products, termed secretome, can be harvested and adopted for cellular-substitution therapy. There are multiple bioactive molecules contained in the secretome [136] such as nucleic acid fragments, growth factors, and extracellular vesicles. Direct delivery of stem cell-derived secretome reboots endogenous repair for pulmonary fibrosis [137]. In addition to secretome, the most broadly explored bioactive agent is extracellular vesicles. Extracellular vesicles mediate cellular interaction by transferring biologically active ingredients. Extracellular vesicles include microvesicles and exosomes, the latter of which has gained much interest in regenerative therapy of cardiac, renal, cerebral and pulmonary diseases [138,139,140]. Exosomes harvested from dermal fibroblast spheroids ameliorated skin photoaging by downregulation of TNF-α and upregulation of TGF-β [141]. Exosomes containing miR-218-5p promote hair regeneration by regulating β-catenin signaling [142]. Inhalation of lung spheroid cell secretome and exosomes has been reported to promote lung repair in pulmonary fibrosis [137]. In the setting of MI, initial studies using human embryonic-derived MSC-exosomes reduced cardiac infarct size in a mouse model of myocardial I/R injury via the activation of the PI3K/Akt signaling pathway, which preserved myocardial viability and inhibited adverse remodeling [143]. In another study, atorvastatin pretreatment enhanced the therapeutic efficacy of MSC-derived exosomes for MI via upregulation of long non-coding RNA H19 [144]. In the exosomes isolated from heart failure patients (FEXO), expression of miR-21-5p is downregulated as compared with a healthy donor, which impaired the ability of FEXO in promoting endothelial tube formation. Restoring miR-21-5p expression rescued FEXO’s reparative function [145]. Moreover, growth factors derived from stem cells, such as bFGF and VEGF are also used for cardiac treatment. Delivery of these growth factors promotes vascular regeneration and improves cardiac remodeling. Taken together, all these studies proved the therapeutic efficacy of direct infusion of acellular components.

The role of stem cell paracrine products in cardiac repair has been studied for decades and has advanced our knowledge in stem cell therapy. However, the paracrine therapeutics may not fulfill the full medical need for heart muscle regeneration in advanced heart failure patients. Therefore, it is most likely that acellular therapeutics would be developed to be one kind of drug used to prevent or slow the progression of disease. Moreover, due to the complexity of paracrine products, clearly understanding the signaling pathways underlying acellular therapy remains a challenge. In addition, there are also problems of targeting, retention, and efficiency, which always need to be addressed during the application of acellular therapy.

## 5. Bioengineering Boosts Acellular Therapy

There is massive accumulation of acellular therapeutic agents in the liver and kidney after intravenous injection, which reveals rapid clearance and compromised efficacy. To optimize acellular therapy, fabrication and modification of bioengineered secretome, exosomes, antibodies, and nucleic acids with elevated targeting features and prolonged retention have been introduced [9,146].

### 5.1. Bioengineered Fabrication of Stem Cell Mimics

**Figure 1:**
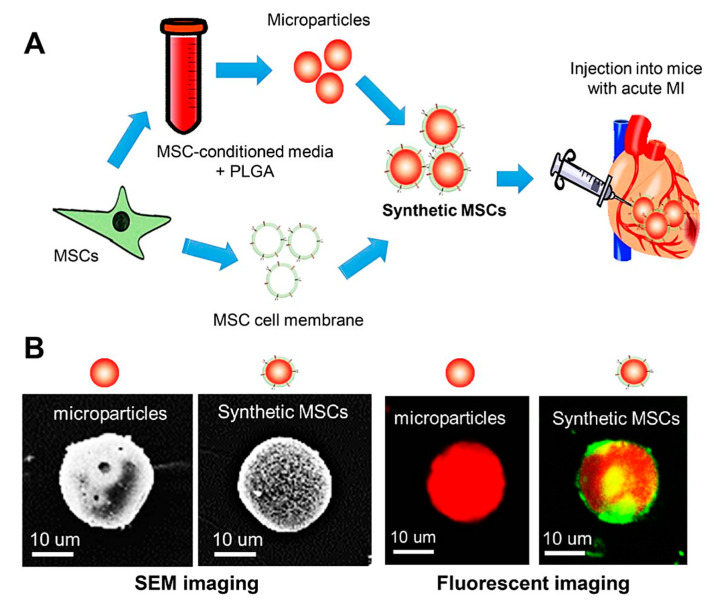
Fabrication and characterization of synthetic mesenchymal stem cell (synMSC). (**A**) Schematic illustration of the fabrication process of synMSC. Microparticles (MP) were fabricated by treating mesenchymal stem cells conditioned media with poly(lactic-co-glycolic acid) (PLGA). Synthetic mesenchymal stem cells (synMSC) were formed by coating the MP with MSC membranes. After that, we tested the therapeutic effects of synMSC injection in mice with acute myocardial infarction; (**B**) Scanning electron microscopy images (left) and fluorescent images (right) of the structure of MP and synMSC. MP were labeled with Texas Red succinimidyl ester (red) and synMSC, as cell membranes were labeled with green fluorescent DiO (red particle with green coat). Reproduced with permission [147].

Nanomedicine is one important aspect of biomedical engineering. Nanoparticles (NPs) have attracted lots of interest in biomedical studies because of their targeting and loading properties. Secretome is the sum of all the products secreted by cells. It can be easily acquired by lyophilizing conditioned medium. To achieve high-efficient delivery, it is reasonable to develop a paradigm to fill secretome into capsule-like materials. Poly(lactic-co-glycolic acid) (PLGA) is an FDA approved biodegradable polymer that has been widely used for nanomedicine preparation. By using PLGA as the carrier, secretome derived from MSCs have been loaded to make therapeutic particles [147] (Figure 1). After infusion, these particles promoted cardiac repair. Moreover, this strategy was also used to fabricate cell-mimicking microparticles (CMMPs), in which cardiac stem cell (CSC)-derived secretome was recoated with CSCs membrane [148]. After intramyocardially injection, CMMPs contribute to the preservation of viable myocardium and augmentation of cardiac functions. The therapeutic efficacy was similar to cardiac stem cell therapy [146]. In addition to the optimization of therapeutic efficacy, construction of the secretome-containing cell mimics increased the stability of paracrine products. By fabrication of an artificial cardiac patch that is loaded with synthetic CSCs (synCSCs), Huang et al. [149] provided a clinically feasible, easy-to-store, off-the-shelf, and cell-free alternative to myocardial repair. In a rat model of acute MI, treatment with synCSCs enhanced cardiac recovery by reducing scarring, promoting angiogenesis, and boosting cardiac function. In a clinically relevant porcine model of MI, the safety and efficacy synCSCs delivery for cardiac repair was confirmed [149].

### 5.2. Bioengineered Exosomes

**Figure 2:**
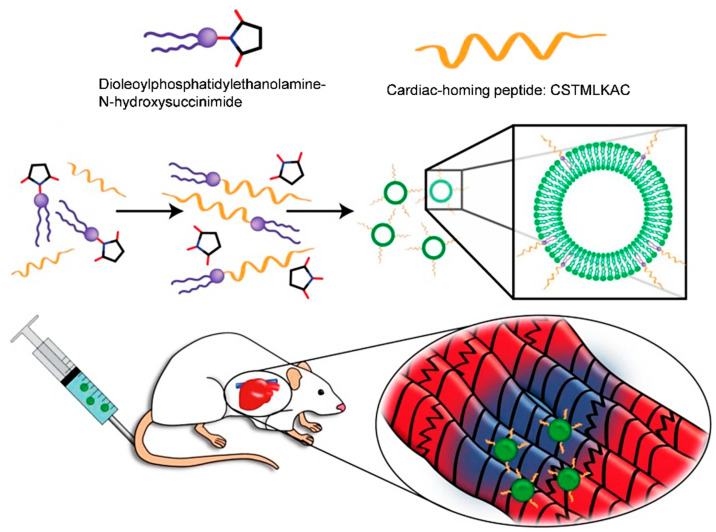
Cardiac-homing exosomes. Myocardium-targeting exosomes were produced by reacting DOPE-NHS to cardiac homing peptide (CHP). Then, the lipophilic tails of the DOPE-CHP spontaneously insert into the exosomal membrane, coating the exosome in CHP peptide. The exosomes were, then, intravenously injected into rats following I/R injury. Reproduced with permission [146].

In addition to secretome, exosomes can also be biomedically modified to have enhanced targeting properties. Exosomes are small vesicles that are released by cells to mediated cell–cell communication. Even though the application of exosomes for cardiac therapy have achieved massive advances, it is worthwhile to note that exosomes are mostly distributed in the liver and kidney after intravenous infusion (i.v.), which may result in undesired side effects and lower therapeutic efficacy. To overcome the drawbacks, modification of exosomes with cardiac homing peptides achieved infarct-targeted accumulation of exosomes after i.v. injection [146]. With the targeted delivery, significant improvement of cardiac function in MI rats was disclosed [146] (Figure 2).

### 5.3. Bioengineered Construction of microRNA Mimics

**Figure 3:**
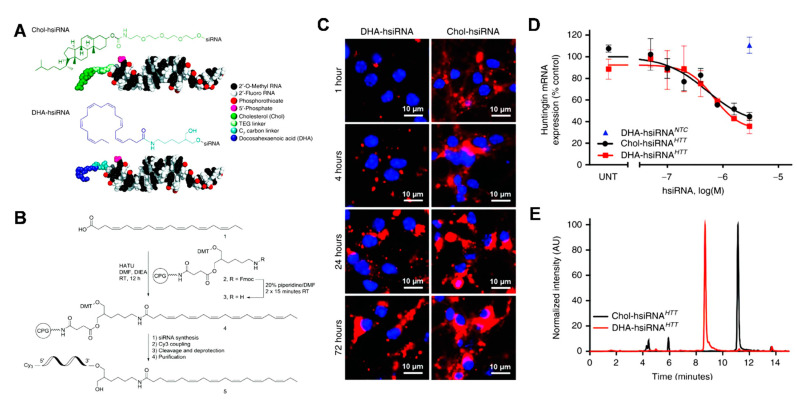
Schematic illustration of the construction of cholesterol-conjugated miRNA. (**A**) Fully chemically stabilized hsiRNA with either docosahexaenoic acid (DHA) or cholesterol (Chol) bioconjugates at the 3′-end of the sense strand; (**B**) Solid-phase synthesis of DHA-conjugated hsiRNA. Htt, Huntingtin; (**C**) Intracellular uptake of Cy3-DHA-hsiRNA and Cy3-Chol-hsiRNA; (**D**) Gene expression detection after hsiRNA incubation; (**E**) High-performance liquid chromatography (HPLC) traces of DHA-hsiRNAHTT and Chol-hsiRNAHTT following reverse phase chromatography. Reproduced with permission [151].

During the exploration of mechanisms underlying the therapeutic effects of exosomes, microRNAs are identified as a population of bioactive molecules that mediate gene expression and modulate cellular activities. MicroRNAs belong to the class of small non-coding RNAs that are involved in the regulation of gene expression. Because the nucleic acid sequence of a known microRNA can be defined, it has increased accessibility of microRNA in pharmaceutic application [150]. Nucleic acids have difficulty crossing the cell membrane to enter the cell. To favor transmembrane delivery, agents such as liposomes, cell penetrating peptide (CPP), and cholesterol [151] are used to construct carriers for microRNAs delivery (Figure 3). Liposomes are widely used for microRNA delivery. When preparing liposomes, functionalized groups can be chemically conjugated to improve the targeting and release behaviors of liposomes. Wang et al. [152] modified miR-302 with cholesterol, a lipid soluble macromolecule. This modification enhanced the internalization of miR-302 by cardiomyocytes and led to improved outcomes after myocardial infarction.

### 5.4. Bioengineered Antibodies for Recruitment of Endogenous Stem Cells

As an alternative to delivery of exogenous stem cells, the recruitment of endogenous stem cells provides an efficient way to repair damaged heart. To target endogenous stem cells, Cheng et al. developed iron nanoparticles chemically modified with two different antibodies, one of which bound to CD34+ cells, and the other targeted cardiomyocytes. Following administration and with the use of an external magnet, the nanoparticles captured endogenous stem cells and transported them to the infarct site [153]. In addition to this strategy, platelet membrane was also used as an infarct-homing carrier [154]. Platelets modified with CD34 antibody on the membrane recruited CD34+ stem cells to the infarcted myocardium [155]. Moreover, it is feasible to synthesize bispecific antibodies (BsAb) to capture both platelet and endogenous stem cells [156].

### 5.5. Challenges and Opportunities for Clinical Translation

Bioengineered acellular therapy provides us with an optimized method to deliver paracrine products. In clinics, intravenous infusion is a common medication administration method. However, direct infusion of paracrine agents has the disadvantage of low retention and lack of targeting. By conjugation of functionalized molecules, the targeting ability of bioengineered acellular therapeutics is enhanced. As a result, the retention rate is elevated, and the therapeutic efficacy is boosted. Bioengineered acellular therapy is most likely to be administrated as a drug. Therefore, it is better to use ischemia/reperfusion MI model in acellular therapy research. Moreover, bioengineered cell mimics have the off-the-shelf feature which makes acellular therapy easily accessible.

However, there are also concerns that need to be addressed before acellular therapy can be applied in clinics. The fabrication, as well as the purification method, need to be well established to generate high amounts of product. Bioengineered agents have to go through overall assessment in terms of biosafety, biodegradation, and biocompatibility. Additionally, the quality control of cell-free products during preparation remains a challenge. Lastly, the precise mechanism needs to be clearly defined.

## 6. Summary and Outlook

Heart diseases are the leading cause of death across the world, accounting for nine million deaths per year in the United States alone. Stem cell therapy and regenerative strategies that pursue the replacement of fibrotic scar with viable cardiac muscle are crucial to control the death rate. Stem cell therapy and biomedical engineering approaches have made heart regeneration feasible. In this review, we summarize promising cell types and underlying mechanisms, as well as the strategies that are potentially efficient in heart regeneration.

Since the heart is not intrinsically regenerative, iPSCs and iCPCs are the most promising cell types because of their differentiation potential to generate cardiomyocytes. Regeneration of cardiac muscle in the infarcted scar is the ideal result from treatment of MI and heart failure patients. However, problems arise with cell therapy because of low engraftment and arrhythmic risks. Due to the intrinsic immune rejection response, the majority of implanted cells do not survive long after implantation. In addition, maturation and coupling with pre-existing myocardium of implanted cells is not successful. As a result, arrhythmia occurs frequently after cell injection. Therefore, cellular therapy for heart diseases will need to be optimized before it can be successfully applied in clinical settings.

**Figure 4:**
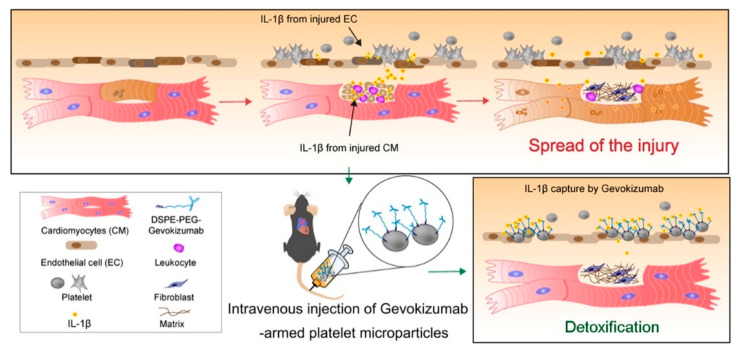
Schematic illustrating the role of Gevokizumab-armed platelet microparticles as cardiac detoxification and repair agents. By taking advantage of the natural infarct-homing abilities of platelet membranes, a platelet-mimicking system that uses anti-IL-1β-neutralizing antibodies was developed in this study. This system functions as an IL-1β decoy that reduces the local inflammatory response in the injured heart in a targeted way. Reproduced with permission [159].

**Figure 5:**
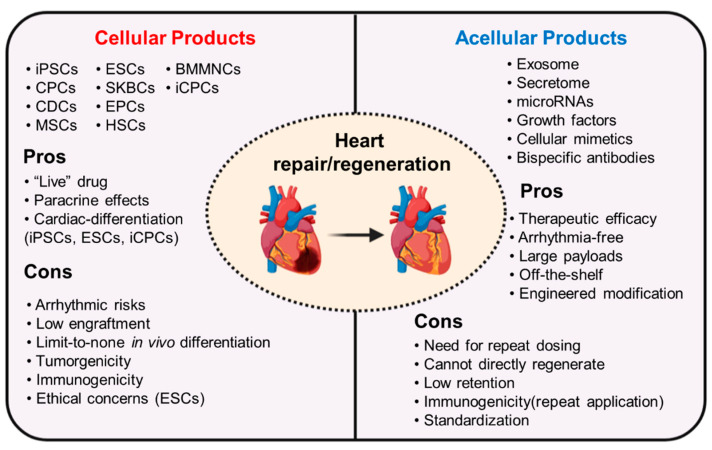
The advantages and disadvantages in cellular and acellular therapy.

In addition to cellular therapy, acellular therapy is most likely to be developed into drugs used for cardiac protection and to suppress the progression of diseases. Paracrine products derived from various types of stem cells, such as growth factors, secretome, exosomes, and miRNAs have been shown to be efficient in improving cardiac histology and ejection function by modulating inflammation, cell proliferation, and remodeling [157,158]. Delivery of these bioactive agents has yielded supporting data for their pharmaceutical value. The concept of acellular therapy has become an important theme in stem cell therapy and regenerative medicine. Moreover, bioengineering techniques have been introduced to overcome drawbacks of direct infusion of acellular therapeutics. Bioengineered modification is advantageous by increasing targeting ability, and thus boosting efficacy of therapy. In addition, bioengineering makes delivery of therapeutics more accessible, for instance, a damage-responsive delivery of paracrine products. Li et al. [159] fabricated platelet microparticles (PMs) armed with anti–IL-1β antibodies, which take advantage of the spontaneous homing properties of platelets towards an injury site. After intravenous injection, the PMs precisely neutralized proinflammatory IL-1β in the injured heart and improved the outcomes of cardiac injury (Figure 4). Another example is the use of reactive oxygen species (ROS) to trigger therapeutics release [158]. However, there are also concerns that need to be addressed when using bioengineered modifications. The advantages and disadvantages of cellular therapy versus acellular therapy are summarized in Figure 5. Some challenges that still remain are that the biosafety and biodegradation of bioengineered products needs to always be assessed. In addition, the quality control for acellular product preparation has yet to be defined. There are numerous biologically active molecules in exosomes, and their levels are easily affected by cell status. Therefore, it is hard to establish a standard for control of exosome quality comparable to that of classical drugs. The precise mechanisms will also need to be defined before acellular therapy can be applied clinically. Despite the challenges, acellular therapy shows therapeutic potentials and holds great pharmaceutic values in the future treatment of heart diseases.

## Data Availability

Data sharing not applicable.
